# Development of a Non-invasive Diagnostic Method for Esophageal Squamous Cell Carcinoma by Gas Chromatographic Analysis of Exhaled Breath

**DOI:** 10.14789/jmj.JMJ22-0023-OA

**Published:** 2022-10-04

**Authors:** SEI MATSUMORI, TAKASHI HASHIMOTO, MOTOMI NASU, NAOKO KAGA, HIKARI TAKA, TSUTOMU FUJIMURA, TAKASHI UENO, YOSHIKI MIURA, YOSHIAKI KAJIYAMA

**Affiliations:** 1 Department of Esophageal and Gastroenterological Surgery, Juntendo University Graduate School of Medicine, Tokyo, Japan; 1 Department of Esophageal and Gastroenterological Surgery, Juntendo University Graduate School of Medicine, Tokyo, Japan; 2 Laboratory of Proteomics and Biomolecular Science, Biomedical Research Core Facilities, Juntendo University Graduate School of Medicine, Tokyo, Japan; 2 Laboratory of Proteomics and Biomolecular Science, Biomedical Research Core Facilities, Juntendo University Graduate School of Medicine, Tokyo, Japan; 3 Laboratory of Bioanalytical Chemistry, Tohoku Medical and Pharmaceutical University, Miyagi, Japan; 3 Laboratory of Bioanalytical Chemistry, Tohoku Medical and Pharmaceutical University, Miyagi, Japan

**Keywords:** Volatile organic compounds (VOCs), Esophageal squamous cell carcinoma (ESCC), Gas chromatography-mass spectrometry (GC-MS)

## Abstract

**Objectives:**

Since esophageal carcinoma progresses asymptomatically, for many patients the disease is already advanced at the time of diagnosis. The main methods that are currently used to diagnose esophageal carcinoma are upper gastrointestinal radiographic contrast examinations and upper gastrointestinal endoscopy, but early discovery of this disease remains difficult. There is a need to develop a diagnostic method using biomarkers that is non-invasive while both highly sensitive and specific.

**Materials and Methods:**

Exhaled breath was collected from 17 patients with esophageal squamous cell carcinoma (ESCC), as well as 9 control subjects without history of any cancer. For each fasting subject, 1L of exhaled breath was collected in a gas sampling bag. Volatile organic compounds (VOCs) were then extracted from each sample using Solid phase micro-extraction (SPME) fibers and analyzed by gas chromatography-mass spectrometry (GC-MS).

**Results:**

Levels of acetonitrile, acetic acid, acetone, and 2-butanone in exhaled breath were significantly higher in the patient group than in the control group (p = 0.0037, 0.0024, 0.0024 and 0.0037, respectively). ROC curves were drawn for these 4 VOCs, and the results for the area-under-the-curve (AUC) indicated that ESCC patients can be identified with a high probability of 0.93.

**Conclusion:**

We found distinctive VOCs in exhaled breath of ESCC patients. These VOCs have a potential as new clinical biomarkers for ESCC. The measurement of VOCs in exhaled breath may be a useful, non-invasive method for diagnosis of ESCC.

## Introduction

Esophageal carcinoma is the tenth most common malignancy in the world^1)^, and the tenth most common cause of cancer death in Japan^2)^. Incidence of squamous cell carcinoma of the esophagus (ESCC) is decreasing in Europe and America, whereas the incidence of esophageal adenocarcinoma is, conversely, trending upward. In Asia, squamous cell carcinoma still accounts for a majority of all esophageal carcinomas^3)^. Known risk factors for ESCC include smoking, drinking alcoholic beverages, hot foods, drinks and malnutrition^4)^. The quality of diagnosis and treatment has improved recently, but these cancers still have a poor prognosis. The main symptoms of esophageal carcinoma are dysphagia and a feeling that food is stuck in the throat. However, because only advanced cancers cause these symptoms, there are usually no symptoms in the early stage of the disease. As a result, discovery of esophageal carcinomas in its early stage remains difficult. Clinical biomarkers and tests for early detection of esophageal carcinomas have not yet been established. Developments of non-invasive method for early diagnosis with better sensitivity and specificity are expected.

In recent decades, various scent detection studies have been performed with animals and electronic devices^5)^. The presence of some infections and malignant tumors can be identified based on changes in patients’ metabolites. Devices for detecting volatile organic compounds (VOCs) that are odorous elements of cancers are also being developed^6, 7)^. McColloch et al. reported high accuracy rates using the exhaled breath of dogs with lung and breast cancers^8)^. Breath analysis using gas chromatography has also identified several VOCs specific to lung and breast cancers^9-11)^. The identification of cancers by exhaled breath analysis is expected to develop as non-invasive methods for detection of early-stage cancer. Recent studies demonstrated the clinical potential of VOCs profiling to identify esophageal or gastric adenocarcinoma^12, 13)^. To date, there have been no reports of VOCs specific to ESCC, and the present pilot study aimed to identify just such VOCs as clinical biomarkers.

## Materials and Methods

### Subjects

Exhaled breath was collected from 17 patients who had been diagnosed with ESCC and 9 healthy volunteers in Juntendo University Hospital between July 2012 and November 2013. Healthy volunteers were selected from staffs at our facility who had been approved to participate in the study and who had no history of cancer or other medical conditions. No upper gastrointestinal endoscopy was required. The exhaled breath was analyzed for its VOC contents. All subjects were interviewed to confirm the disease for which they were being treated, whether they were taking any medications or smoked, and their family histories. Patients who were being treated with either radiotherapy or chemotherapy were excluded from the study, since such treatments might alter their metabolism. All protocols were approved by the ethical committee of the Juntendo University Hospital (No.16-062), and all participants provided written informed consent before their participation in this study according to the guidelines established in the Declaration of Helsinki.

### Exhaled breath collection

All participants were fasted for 6-8 hours prior to their breath sample collection. For each subject, 1L of exhaled breath was collected in a Supel™ Inert Gas Sampling Bag (Sigma-Aldrich Co. LLC. MO, USA) in a clean-air environment before any treatment. Prior to the collection, each sampling bag was washed with nitrogen to reduce other influences. All collected samples were stored and processed in a cold room at 4℃.

### Extraction and analysis of volatile organic compounds

Samples were analyzed by gas chromatography- mass spectrometry (GC-MS) combined with solid phase micro extraction (SPME) (Sigma-Aldrich Co. LLC. MO, USA). The SPME was used for pre- concentration of VOCs in the breath samples. A manual SPME holder with 85μm Carboxen^®^/Polydimethylsiloxane (CAR/PDMS) fiber (Sigma- Aldrich Co. LLC. MO, USA) was inserted into the Supel™ Inert Gas Sampling bags for 16 hours at room temperature. The VOCs extracted in SPME fiber were desorbed thermally in the heated GC injector at 250°C (splitless mode) and analyzed by GC-MS. The GC-MS analysis was performed with a TRACE GC connected to TSQ QUANTUM GC (Thermo Fisher Scientific Inc., MA, USA). A CP Pora PLOT Q capillary column (0.25mm i.d x 25m x 8μm firm thickness, Agilent Technologies Inc, CA, USA) was used for separation of VOCs. The column temperature started at 40°C for 3 minutes, then raised at 10°C per minute to 250°C and held for 5 minutes. Helium was used as carrier gas at 1ml/min. MS transfer line was set at 250°C. The MS analysis was carried out Electron impact ionization (EI) mode with 70eV of electron energy at scan range m/z:10-400. Ion source temperature was set at 250°C. The detected VOCs were identified by NIST MS Search 2.0 (Thermo Fisher Scientific Inc., MA, USA).

### Statistical analysis

After alignment the comprehensive peaks in GC-MS chromatograms of every sample using Thermo Scientific SIEVE Software (Thermo Fisher Scientific Inc., MA, USA), the Fisher Ratio was calculated against individual frames and significant peaks were extracted to find candidate for biomarkers which discriminated 2 groups^14)^. VOCs were identified by comparing EI mass spectra of each peak with that of the NIST Mass Spectral Library Version 2.0f using NIST MS Search 2.0. The area of selected ion peak in each candidate was applied to principal component analysis (PCA). And those of them that were significantly higher in the patient group than in the healthy volunteer group were used to generate receiver-operating characteristics (ROC) curves using the free ROC analysis software (WROCFIT). Statistical analysis was performed using *t*-test. The differentially expressed compounds with p-values of < 0.05 were considered statistically significant. Receiver-operating characteristics (ROC) curve was used to evaluate the discriminatory power of selected VOCs for differentiation of patients and controls.

## Results

### Study population

The clinical characteristics of the patient group are summarized in Table 1. Seventeen esophageal carcinoma patients and 9 volunteer control subjects were enrolled in this study. All subjects in the patient group had been pathologically diagnosed with advanced ESCC.

**Table 1:** Clinical characteristics of esophageal carcinoma patients in this study

Patient no.	Sex	Age	Smoking	Family history of cancer	Histology^a^	Main location of the lesion^b^	Size (mm)	Differentiation^c^	Depth of tumor invasion	Lymph node metastasis	Distant metastasis	Stage
1	M	40	Yes	No	SCC	CeUt	50	WD	T3	N2	No	ⅢB
2	F	66	No	No	SCC	Lt	70	MD	T3	N2	No	ⅢB
3	M	70	Yes	No	SCC	Ce	45	PD	T3	N3	No	ⅢC
4	M	66	Yes	Yes	SCC	Lt	44	MD	T3	N0	lung	Ⅳ
5	M	55	No	Yes	SCC	MtUt	42	MD	T4b	N2	No	ⅢB
6	M	55	Yes	Yes	SCC	Mt	36	MD	T3	N2	No	ⅢB
7	M	67	Yes	Yes	SCC	CeUt	50	MD	T3	N2	No	ⅢB
8	M	66	Yes	Yes	SCC	Mt	39	PD	T2	N2	No	ⅢB
9	M	81	Yes	No	SCC	MtUt	66	MD	T4b	N3	No	ⅢC
10	M	55	Yes	Yes	SCC	Ut	30	PD	T2	N2	No	ⅢB
11	M	62	Yes	Yes	SCC	LtAe	45	MD	T3	N1	No	ⅢA
12	M	70	Yes	Yes	SCC	CeUt	80	MD	T3	N3	No	ⅢC
13	M	65	Yes	Yes	SCC	CeUt	60	MD	T4b	N3	No	ⅢC
14	F	65	Yes	No	SCC	LtMt	76	MD	T3	N3	No	ⅢC
15	M	68	Yes	No	SCC	MtLt	60	MD	T3	N2	No	ⅢB
16	F	83	Yes	No	SCC	Ce	20	MD	T4b	N2	No	ⅢC
17	F	80	No	Yes	SCC	Mt	25	MD	T3	N1	No	ⅢA

^a^ SCC, esophageal squamous cell carcinoma ^b^ Ce, cervical esophagus; Ut, upper thoracic esophagus; Mt, middle thoracic esophagus; Lt, lower thoracic esophagus; Ae, abdominal esophagus ^c^ WD, well differentiated; MD, moderately differentiated; PD, poorly differentiated

### VOCs profiling of samples

**Figure 1:**
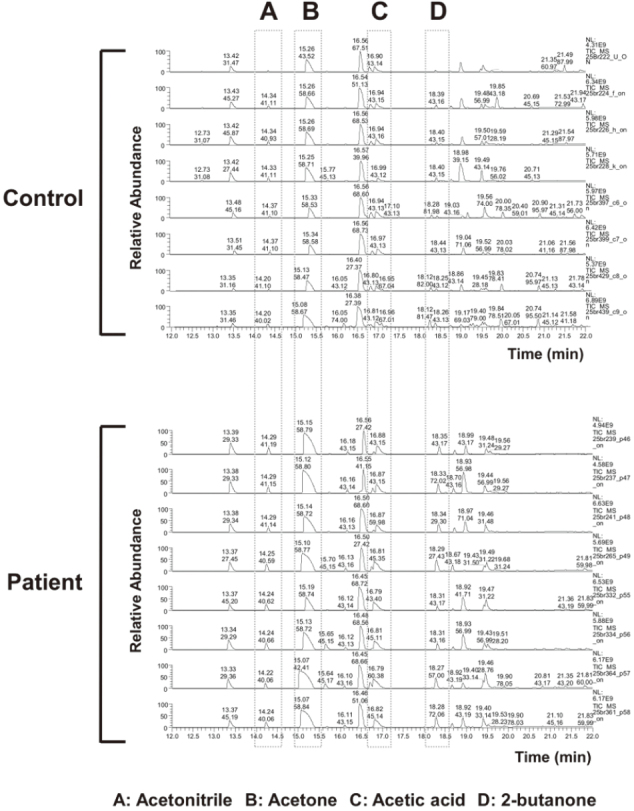
Representative GC/MS total ion chromatograms of the breath samples from the control group and the patient group Each peak shows (A) acetonitrile, (B) acetic acid, (C) acetone, and (D) 2-butanone.

Representative GC/MS total ion chromatograms of samples from the patient group and the control group were showed in Figure 1. Statistical analysis of the obtained peaks revealed that 15 VOCs containing 4 VOCs, i.e. acetonitrile, acetic acid, acetone, 2-butanone, of which were significantly higher in the patient group than in the healthy volunteer group.

**Figure 2:**
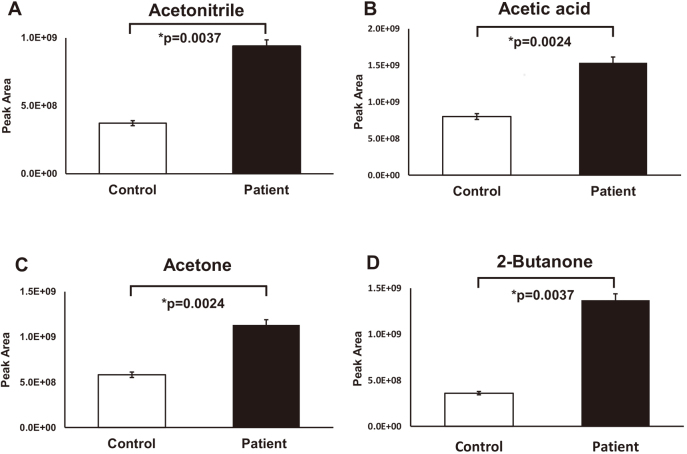
Comparison of the 4 VOCs level between in the control group and the patient group The peak area of (A) acetonitrile, acetic acid, (B)acetone, (C) acetone, and (D) 2-butanone in the ESCC patient group were significantly higher than those in the control group (p<0.01, respectively).

Figure 2 showed differences of peak area for 4 VOCs in two groups. These differences were found to be significant (acetonitrile, p = 0.0037; acetic acid, p = 0.0024; acetone, p = 0.0024; 2-butanone, p = 0.0037).

**Figure 3:**
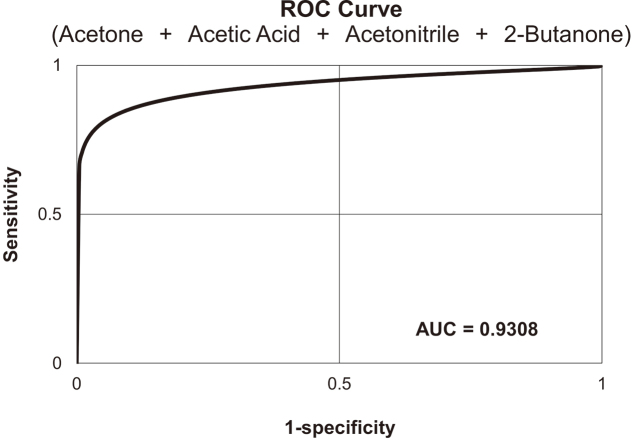
Receiver operating characteristic (ROC) curve for the detection of ESCC patients using the combination of acetonitrile, acetic acid, acetone, and 2-butanone concentration in exhalation The area-under-the-curve (AUC) was 0.93.

ROC curves were drawn for acetonitrile, acetic acid, acetone, and 2-butanone. The results for the area-under-the-curve (AUC) of the combination of these 4 VOCs indicated that ESCC patients can be identified with a high probability of 0.93 (Figure 3).

## Discussion

There was significant difference in VOCs included in exhaled breath between the ESCC patient group and the control group: acetone, acetic acid, acetonitrile, and 2-butanone showed higher concentration in the patient group. To the best of our knowledge, this is the first report that demonstrates the significance of VOCs measurement in ESCC.

Recent years, several VOCs detection by breath analysis with GC/MS have been reported to be useful to identify lung and breast cancers^8-10)^. Metabolomics studies for detecting various cancers are increasing^15-17)^. However, studies that focus on ESCC are still relatively rare. Cancer growth is promoted by mutations in genes and proteins, leading to peroxidation of cell membranes and release of VOCs^18)^. Endogenous cancer-specific VOCs are released from the cancer cells themselves and/or during the metabolic processes associated with cancer growth, and it is suspected that different substances and/or compounds with different molecular weights are released by different types of cancers. Moreover, it is not just that VOCs are released directly from cancers, but also that metabolites are conveyed from the blood stream by alveoli and enter the exhaled breath^19-21)^. As a result, it is thought that it is possible to measure the changes in metabolism that are caused by cancer growth in exhaled breath^22)^.

In esophageal carcinoma patients, the exhaled breath is thought to include not only metabolites originating from the alveoli, but also many fragrant substances arising from the esophageal carcinoma itself. Thus, it is thought that breath analysis of VOCs would make it possible to identify substances specific to ESCC more accurately compared with other cancers. Altomare et al. found that 15 VOCs, including alkyl aldehydes, ketones, alkanes, and aromatic hydrocarbons, differed between healthy subjects and patients with colorectal cancer^23)^. Huang et al. measured VOCs in urine samples and found differences in acetone, acetic acid, hexane, hydrogen sulfide, methanol, and phenol between patients with gastro esophageal cancer and healthy subjects^24)^. Kumar et al. demonstrated that 12 VOCs in exhaled breath were present at significantly higher concentrations in the esophageal and gastric adenocarcinoma cancer groups than in the noncancer controls^13)^. They reported that VOCs profiling was useful for early detection of cancers.

The present study has several limitations. First, this was a single center study with a small sample size, and it will be necessary to study a larger number of samples to confirm these findings. Second, the patients in this study had comparatively advanced cancers. There have been reports that lung, colorectal, and breast cancers could be distinguished from healthy individuals at a relatively early stage using this method^25-27)^. Therefore, this method of cancer detection must be tested in patients with early-stage ESCC. Third, there are no established methods for breath analysis of VOCs, and there is a need to evaluate whether food, tobacco use, ingested drugs, concurrent diseases, genetic factors, etc., affect the results of this analysis method. Moreover, the composition of gastrointestinal microbiota could have a major impact on the exhaled breath of suspected digestive cancer patients; this factor has not yet been studied sufficiently in the context of diagnostic breath testing^28)^.

In conclusion, this study found that the levels of 4 VOCs, i.e., acetonitrile, acetic acid, acetone, and 2-butanone, were significantly higher in the exhaled breath of ESCC patients than in control subjects. Those findings indicate that measurement of VOCs in the exhaled breath has potential as a useful, non invasive method for diagnosis of ESCC.

## Funding

No funding was received.

## Author contributions

SM and YK made a study design, recruited patients and volunteers as control, collected data, analyzed, and interpreted the patient data and SM was a major contributor in writing the manuscript. NK, HT, TF, and TU performed the examination of the gas chromatography-mass spectrometry (GC- MS). TH and MN revised the article, and all authors approved the final manuscript.

## Conflicts of interest statement

The Authors declare that there are no conflicts of interest.
